# Effect of Photoperiod on Ascorbic Acid Metabolism Regulation and Accumulation in Rapeseed (*Brassica napus* L.) Seedlings

**DOI:** 10.3390/antiox14020160

**Published:** 2025-01-29

**Authors:** Chao Wang, Lieqiong Kuang, Ze Tian, Xinfa Wang, Jinxing Tu, Hanzhong Wang, Xiaoling Dun

**Affiliations:** 1 Key Laboratory of Biology and Genetic Improvement of Oil Crops of the Ministry of Agriculture and Rural Affairs, Oil Crops Research Institute of the Chinese Academy of Agricultural Sciences, Wuhan 430062, China; wangchao05360536@163.com (C.W.); kuanglieqiong@163.com (L.K.); tianze0825@163.com (Z.T.); wangxinfa@caas.cn (X.W.); 2 National Key Laboratory of Crop Genetic Improvement, Huazhong Agricultural University, Wuhan 430070, China; tujx@mail.hzau.edu.cn

**Keywords:** ascorbic acid, rapeseed seedlings, photoperiod, enzyme activity, gene expression

## Abstract

Ascorbic acid (AsA) is an important antioxidant for human health. The concept of “oil-vegetable-duel-purpose” can significantly enhance the economic benefits of the rapeseed industry. Rapeseed, when utilized as a vegetable, serves as a valuable food source of AsA. In this study, we integrated transcriptome and metabolome analyses, along with substrate feeding, to identify the L-galactose pathway as the primary source for AsA production, which is primarily regulated by light. Through seven different photoperiod treatments from 12 h/12 h (light/dark) to 24 h/0 h, we found that AsA content increased with longer photoperiods, as well as chlorophyll, carotenoids, and soluble sugars. However, an excessively long photoperiod led to photooxidative stress, which negatively affected biomass accumulation in rapeseed seedlings and subsequently impacted the total accumulation of AsA. Furthermore, different enzymes respond differently to different photoperiods. Analysis of the correlation between the expression levels of AsA biosynthesis-related genes and AsA content highlighted a dynamic balancing mechanism of AsA metabolism in response to different photoperiods. The study revealed that the 16 h/8 h photoperiod is optimal for long-term AsA accumulation in rapeseed seedlings. However, extending the photoperiod before harvest can enhance AsA content without compromising yield. These findings offer novel insights into an effective strategy for the biofortification of AsA in rapeseed.

## 1. Introduction

Ascorbic acid (AsA), commonly known as vitamin C (Vc), is a widely recognized water-soluble vitamin that is essential for both humans and animals, playing an indispensable role in their antioxidant systems [1,2]. Many research studies have emphasized the significance of AsA in maintaining health and preventing oxidative stress-related illnesses, including various types of cancers, cardiovascular diseases, and the effects of aging, among other health concerns [3,4,5,6]. However, humans have lost the function of the gene coding for L-gulonolactone oxidase, the enzyme responsible for catalyzing the final step in the AsA synthesis pathway, and thus cannot synthesize AsA endogenously, relying instead on dietary sources such as fresh fruits and vegetables to meet their daily requirement [7,8]. Overall, the current dietary guidelines recommend daily consumption of AsA between 40 mg and 110 mg in different regions of the world, a variation that reflects the recognition of AsA’s importance in maintaining optimal health and its varying requirements among different populations [9]. The market demand for AsA continues to grow worldwide, highlighting its significance across various sectors and the rising consumer awareness of its health benefits [10]. Therefore, improving the AsA content in vegetables is crucial for enhancing the commodity value of vegetables and sustaining human health.

AsA is acknowledged as a vital antioxidant found in plants, exhibiting substantial reactive oxygen species (ROS) scavenging potential, and plays a vital role in various physiological processes within plant systems, including growth and development, photosynthesis, metabolic processes, hormone signaling, and resistance to various environmental stresses [11,12,13,14]. As previously reported, four biosynthesis pathways for AsA in plants have been identified by researchers, including the L-galactose (D-mannose), L-gulose, Myo-inositol, and D-galacturonate pathways [15]. Among the pathways identified for AsA synthesis in plants, the L-galactose pathway is recognized as the most significant, which is characterized by some intricate enzyme-catalyzed reactions mediated by several essential enzymes, including GDP-D-mannose pyrophosphorylase (GMP), GDP-L-galactose phosphorylase (GPP), and L-galactono-1,4-lactone dehydrogenase (GLDH) [16,17]. GMP serves as the crucial initial enzyme of AsA, facilitating the formation of GDP-D-mannose, a vital intermediate in the production of AsA [18]. GGP catalyzes the conversion of GDP-L-galactose into L-galactose-1-phosphate [19]. GLDH catalyzes the last stage of the AsA synthesis, which helps oxidize L-galactose-1,4-lactone, GLDH catalyzes the last stage of the AsA synthesis, which helps oxidize L-galactose-1,4-lactone, ultimately leading to the production of AsA [20]. Additionally, the recycling pathway of AsA, which significantly influences the total AsA content within plants, involves several essential enzymes, including monodehydroascorbate reductase (MDHAR), dehydroascorbate reductase (DHAR), ascorbate oxidase (AAO), and ascorbate peroxidase (APX).

In recent years, many countries and regions have vigorously developed horticulture facilities, using modern agricultural facilities to achieve the large-scale, automated, and anti-seasonal production of high-value products and improve agricultural production efficiency and economic benefits. In plant factories, leafy vegetables have become a primary choice for hydroponic cultivation due to their quick growth, compact form, and high demand in the market [21]. In controlled plant factories, by optimizing the temperature, light exposure, carbon dioxide concentration, and growth media, photosynthesis and nutrient absorption of plants can be enhanced, thereby improving the yield and nutritional value of vegetables. Understanding how phytonutrients interact with the horticultural environment is fundamental to obtaining high-nutrient products. Light plays an essential role in controlling phytodevelopment and vegetative growth, affecting gene expression and enzymatic activity within the pathways of AsA biosynthesis and recycling pathways [22,23,24,25]. Research indicates that the production of AsA is directly linked to photosynthesis and is influenced by the quality and intensity of light and duration of light exposure [26,27,28]. *Arabidopsis* leaves showed increased expression of phosphomannose isomerase 1 (*PMI1*) and higher AsA levels under continuous light conditions [29]. GDP-mannose-3′, 5′-epimerase 1 (*GME1*) is significant in the biosynthesis of AsA in tomatoes, and its expression and activity are modulated by light [23]. Enzymatic activities and gene expression related to *GLDH* have decreased in *Arabidopsis* [30] and Chinese cabbage [31] when the leaves were exposed to shade or darkness.

Rapeseed is recognized as the third-largest oilseed crop globally, which is the most significant oilseed crop in China [32,33]. *Brassica napus* (AACC, 2n = 38) is an allopolyploid hybrid species created through the distant crossing of *B. rapa* (AA, 2n = 20) and *B. oleracea* (CC, 2n = 18) [34]. The established popularity of *B. rapa* and *B. oleracea* as consumed vegetables supports the scientific foundation for *B. napus* to be cultivated as a high-potential vegetable. Moreover, “oil-vegetable-duel-purpose” serves as a significant illustration of the multi-functional application of rapeseed to enhance industrial efficiency in China [35,36]. In our earlier studies, we discovered that rapeseed seedlings and flower stalks contain high levels of AsA, which is beneficial as a food source [37]. However, the regulatory mechanisms of AsA biosynthesis in rapeseed and its interaction with environmental factors are still not clear. The objective of this research creatively aimed to identify the primary synthetic pathways influencing AsA in rapeseed and to determine how photoperiod affects AsA levels through the analysis of biosynthetic pathways, key enzyme activities, and expression levels of key genes during rapeseed growth. This study provides theoretical support for the establishment of a rapeseed high-AsA ecological cultivation system, which is conducive to improving the commercial worth of rapeseed products and fostering the rapeseed industry’s development.

## 2. Materials and Methods

### 2.1. Plant Material

Previous studies conducted by the Rapeseed Genetics and Breeding Innovation Team at the Oil Crop Research Institute of the Chinese Academy of Agricultural Sciences (Wuhan, China) have established a natural *B. napus* population characterized by extensive genetic variation [38]. In this study, six high-AsA accessions (8S079, 8S136, 8S183, 8S200, 8S242, and 8S243) and six low-AsA accessions (8S007, 8S027, 8S046, 8S084, 8S132, and 8S158) were selected from the natural population. For the cultivation of rapeseed, we adopted greenhouse hydroponic experiments previously published by our team, which provides a low-cost and high-efficiency, ensuring consistent and reproducible experimental conditions [39,40]. The seeds were sown on medical gauze within the germination device for 2 days in darkness, followed by growth under light conditions (180 µmol·m^−2^·s^−1^, 16 h/8 h, light/dark) for 4 days in a greenhouse maintained at 24 ± 2 °C with 60–80% relative humidity. Six days after sowing (6DAS), consistent and vigorous seedlings were selected and transplanted into the growth setup with a 25% Hoagland solution. After a subsequent week, the solution was substituted with a 50% solution, followed by weekly replacements with a 100% solution until harvest.

### 2.2. Substrate Treatment Experiment

At 14DAS, we conducted a substrate treatment experiment on rapeseed accessions that exhibited varying levels of AsA (8S007, 8S242, and 8S243). Using several substrates associated with the AsA synthesis pathway, including glucose, D-mannose, L-galactose, and L-galactose-1,4-lactone corresponding to the D-mannose (L-galactose) pathway, inositol, D-glucurone corresponding to the inositol pathway, D-galacturonic acid corresponding to the D-galacturonate pathway, and L-gulonic acid-1,4-lactone corresponding to the (L-galactose) pathway, inositol, D-glucurone corresponding to the inositol pathway, D-galacturonic acid corresponding to the D-galacturonate pathway, and L-gulonic acid-1,4-lactone corresponding to the L-gulose pathway, all at a concentration of 50 mM. Additionally, ddH_2_O was used as the control. After continuous spraying for 4 days, with each spray volume set at 20 mL, samples were collected from the above-ground tissue to determine AsA content.

### 2.3. Different Photoperiod Treatment Experiments

The high-AsA accession (8S243) of rapeseed seedlings underwent a 10-day acclimation period under uniform light intensity and a consistent photoperiod of 16 h/8 h (light/dark). Following acclimation, the rapeseed seedlings were subjected to different photoperiod treatments, specifically set to 12 h/12 h, 14 h/10 h, 16 h/8 h, 18 h/6 h 20 h/4 h, 22 h/2 h, and 24 h/0 h. Rapeseed seedlings were collected after being grown under different photoperiods for 1, 2, 3, and 4 days. The period from planting the materials to completion of the photoperiod treatment was from 1 to 20 March 2024. Three biological replicates were harvested, swiftly frozen with liquid nitrogen, pulverized into a fine powder, and subsequently kept at −80 °C for subsequent analysis.

### 2.4. Determination of AsA Content

The measurement of AsA content was performed following the method as previously described [37]. Frozen rapeseed seedlings were finely ground, and 1.0 g of the resulting powder was accurately measured and extracted using 25 mL of 0.1% hydrochloric acid. The resultant mixture underwent filtration through a 0.22 μm hydrophilic polytetrafluoroethylene syringe filter membrane, yielding a clear supernatant. The conditions of the HPLC-PDA system (Waters, Milford, MA, USA) were established as follows: a C18 column (250 mm × 4.6 mm × 5 μm, Waters, USA); a flow rate maintained at 1.0 mL/min; an injection was set at a volume of 10 μL; a target temperature for the column was maintained at 25 °C; a detection wavelength of 245 nm; a retention time for AsA measured at 2.7 min; the mobile phase was composed of a mixture of methanol and 20 mM ammonium acetate in a ratio of 3:97 (*v*/*v*). To ensure accurate quantification of AsA content in rapeseed seedlings, reference standard curves for AsA were constructed.

### 2.5. Determination of ROS-Related Indicators (H_2_O_2_, O^2−^, and MDA Content)

The hydrogen peroxide (H_2_O_2_), superoxide anion radical (O^2−^), and malondialdehyde (MDA) were measured using a commercial kit purchased (Solarbio, Beijing, China). The experimental steps were carried out strictly according to the kit instructions, and the experiments included three independent biological repetitions (mean ± SD, n = 3). All spectrophotometric measurements were performed using an ultramicro microporous plate spectrophotometer (BioTek Epoch, Santa Clara, CA, USA).

### 2.6. Determination of Soluble Sugars Content

The content of soluble sugars was determined by anthrone sulfuric acid colorimetry [41]. Approximately 0.1 g of finely ground sample powder was mixed with 1 mL of distilled water to grind. This resultant mixture was incubated for 10 min in a water bath maintained at 100 °C, followed by cooling and centrifugation at 10,000 rpm for 10 min at 25 °C. The supernatant was then meticulously moved to a 10 mL tube, diluted to reach a final volume of 10 mL with additional distilled water, thoroughly shaken, and set aside. Next 20 µL of the anthraquinone working solution was added to the test tube, along with 80 µL of distilled water and 200 µL of concentrated sulfuric acid, mixed well and incubated for 10 min in a water bath at 95 °C. After the reaction product cools down, carefully transferred 200 µL of the upper aqueous phase to 96-well plates. Finally, the absorbance of the resulting solution was measured at a wavelength of 620 nm, and the concentration of soluble sugars was determined by referencing a previously established standard curve. The experiments were performed with three independent biological repetitions (mean ± SD, n = 3). All spectrophotometric measurements were performed using an ultramicro microporous plate spectrophotometer (BioTek Epoch, USA).

### 2.7. Measurement of Chlorophyll and Carotenoids Content

The content of chlorophyll and carotenoids was determined by referring to previous methods using spectrophotometric analyses [42,43]. Approximately 100 mg of finely ground rapeseed seedlings powder was combined with 20 mL of 95% ethanol and incubated in the dark at 25 °C for 24 h to facilitate extraction. Absorbance readings of the supernatant were taken at wavelengths of 665 nm, 649 nm, and 470 nm using a spectrometer, and the experiments included three independent biological repetitions (mean ± SD, n = 3). All spectrophotometric measurements were performed using an ultramicro microporous plate spectrophotometer (BioTek Epoch, USA).

The total chlorophyll concentration was determined using the following formulas:

C_a_(Chlorophyll a, mg/g FW) = 13.95 × OD_665_ − 6.88 × OD_649_

C_b_(Chlorophyll b, mg/g FW) = 24.96 × OD_649_ − 7.32 × OD_665_

Total Chl (Total chlorophyll, mg/g, FW) = C_a_ + C_b_

Carotenoids concentration was determined using the following formulas:

Car(Carotenoids, mg/g FW) = (1000 × OD_470_ − 2.05 × C_a_ − 114.8 × C_b_)/245

### 2.8. Determination the Activities of Enzymes

The activities of the enzymes GGP and GMP were determined by a commercial kit purchased (Enzyme-linked, Shanghai, China). Meanwhile, the activities of the enzymes GLDH, DHAR, MDHAR, and APX were determined by a different commercial kit purchased (Solarbio, China). The experimental steps were carried out strictly according to the kit instructions with three biological repetitions (mean ± SD, n = 3). All spectrophotometric measurements were performed using an ultramicro microporous plate spectrophotometer (BioTek Epoch, USA).

### 2.9. RNA Extraction and Quantitative Real-Time PCR

Total RNA was extracted from the leaves of rapeseed seedlings using an RNA Extraction Kit (Omega, Macon, GA, USA), and cDNA was synthesized using the HiScript^®^ III All-in-one RT SuperMix Perfect (Vazyme, Nanjing, China). Quantitative real-time PCR (qRT-PCR) was conducted using the ChamQ Universal SYBR qPCR Master Mix (Vazyme, China). The qRT-PCR analysis was accomplished by LightCycler^®^ 480 Ⅱ Real-Time PCR System (Roche, Basel, Switzerland). The 2^−ΔΔCt^ method approach was utilized to determine the relative transcript levels of the targeted gene, and three biological replicates were performed for each qRT-PCR experiment. *ACTIN7* was selected as the reference gene to normalize the expression data. A complete list of all primer sequences utilized throughout this research can be found in Supplemental Table S1.

### 2.10. RNA Sequencing and Data Analysis

At 6DAS and 12DAS, leaves from six high-AsA and six low-AsA rapeseed accessions were collected and mixed in equal amounts to form four AsA mixed pools (H6DAS, L6DAS, H12DAS, and L12DAS). To ensure reliability and accuracy in the analysis, each mixed pool contained three biological replicates. The construction of mRNA libraries and subsequent sequencing of these samples were executed on the BGISEQ-500 platform. The resulting clean data from the sequencing process were aligned to the reference genome of ZS11.v0 [44] as well as gene sequences using the HISAT comparison software [45] and Bowtie2 [46]. Differentially expressed genes (DEGs) were detected using the specific statistical thresholds of *p*-value ≤ 0.05 and |log_2_(fold change)| ≥ 1.

### 2.11. Metabolome Extraction and Data Analysis

The samples previously mentioned were also utilized for metabolome analysis. Six biological replicates of each AsA pool were sent to BGI (Wuhan, China) for LC-MS analysis. The data cleaning, statistical evaluations, metabolite sorting, and functional annotations were all conducted utilizing the BGI’s metabolomics software, known as metaX [47]. The established criteria for screening included a Fold-Change ≥ 1.2 or ≤0.83, a *q*-value < 0.05, and VIP values from the first two principal components of the PLS-DA model ≥ 1, which were utilized to ascertain the differentially accumulated metabolites (DAMs) across samples [48,49].

### 2.12. Statistical Analysis

To ensure the accuracy and credibility of the findings, statistical analyses were conducted with a minimum of three replicates per experiment or biological sample. In the figures, the different letters indicate the significance groupings (*p* < 0.05), as determined by ANOVA using SPSS software (IBM SPSS Statistics 26, Chicago, IL, USA), and error bars represent standard deviations. Additionally, principal component analysis (PCA) was conducted using the ‘PCA’ function available in the Origin software (2024b, Origin Laboratory, Northampton, MA, USA).

## 3. Results

### 3.1. Transcriptome and Metabolome Analysis Revealed the Association Between Photosynthesis and AsA Synthesis in Rapeseed

**Figure 1:**
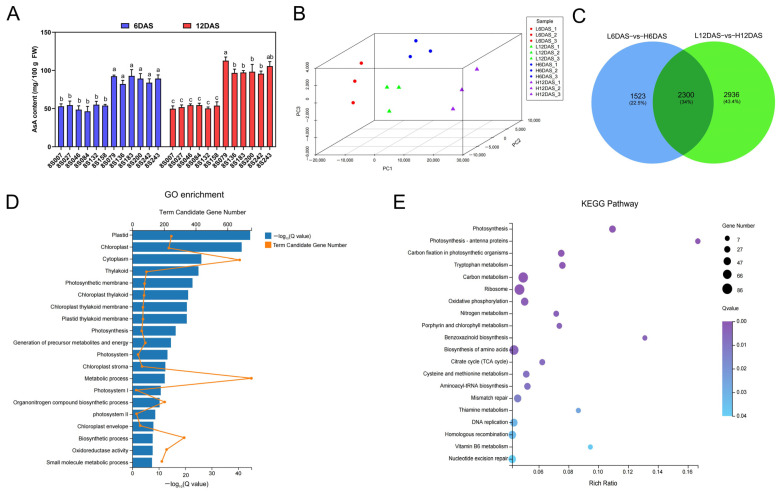
Overview of transcriptome analysis. (**A**) Comparison of AsA content of high and low AsA accessions at 6DAS and 12DAS. (**B**) PCA of gene expression for high- and low-AsA mixed pools. (**C**) Venn graph for DEGs from L6DAS-vs-H6DAS and L12DAS-vs-H12DAS. (**D**) The GO enrichment analysis of overlapped DEGs. (**E**) The KEGG pathway enrichment analysis of overlapped DEGs. The different letters above the bars denote significance groupings (*p* < 0.05) as determined by ANOVA. FW, fresh weight.

In order to identify the primary pathway of AsA synthesis in rapeseed, the high- and low-AsA mixed pools with 6 accessions each at 6DAS and 12DAS were selected for transcriptome and metabolome analysis (Figure 1A). For transcriptome data, three biological replicates were established. Each sample obtained at least 6 Gb of clean sequencing data, which were successfully aligned to the ZS11 reference genome with alignment values higher than 89% (Table S2). The expression patterns of 12 randomly selected genes from the DEGs detected by qRT-PCR were generally consistent with those obtained through the RNA-seq method, thereby confirming the reliability of the RNA-seq results (Figure S1). The correlation analysis (Figure S2) and PCA (Figure 1B) demonstrated good biological repeatability and clear differences between the high- and low-AsA mixed pools. Among the comparisons of L6DAS-vs-H6DAS and L12DAS-vs-H12DAS, 2300 DEGs were found to be overlapped (Figure 1C). Further, GO enrichment analyses suggested that these DEGs were enriched in terms of their association with plastid, chloroplast, cytoplasm, thylakoid, photosynthetic membrane, chloroplast thylakoid, chloroplast thylakoid membrane, plastid thylakoid membrane, photosynthesis and generation of precursor metabolites and energy (Figure 1D). The DEGs were significantly enriched in several KEGG pathways, including those related to photosynthesis, photosynthesis-antenna proteins, carbon fixation in photosynthetic organisms, tryptophan metabolism, carbon metabolism, ribosome, oxidative phosphorylation, nitrogen metabolism, porphyrin and chlorophyll and benzoxazinoid biosynthesis (Figure 1E). Overall, most DEGs were closely related to photosynthesis, energy metabolism or photosynthetic organelles.

**Figure 2:**
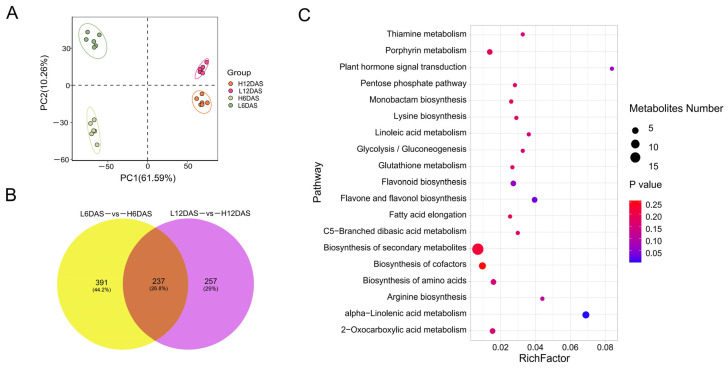
Overview of metabolome analysis. (**A**) PCA of metabolites for AsA mixed pools. (**B**) Venn graph for DAMs from L6DAS-vs-H6DAS and L12DAS-vs-H12DAS. (**C**) The KEGG pathway enrichment analysis of overlapped DAMs.

Metabolome identification was performed using the above samples, including six biological replicates. The PCA chart illustrated the evident separation of the metabolites in the four AsA pools (Figure 2A). Comparisons between L6DAS-vs-H6DAS and L12DAS-vs-H12DAS identified 628 and 494 DAMs, respectively, with 237 DAMs overlapping (Figure 2B). In addition, these DAMs were chiefly enriched in the KEGG pathways such as the biosynthesis of secondary metabolites, biosynthesis of cofactors, alpha-linolenic acid metabolism, porphyrin metabolism, 2-oxocarboxylic acid metabolism, biosynthesis of amino acids, flavonoid biosynthesis, flavone and flavonol biosynthesis (Figure 2C). Among these, the porphyrin metabolism pathway, which is the basis for chlorophyll biosynthesis and functional maintenance in photosynthesis [50], was also analyzed and enriched in DEGs by transcriptomics. Transcriptome and metabolome analysis showed that AsA metabolism was highly correlated with photosynthesis, energy metabolism, and chlorophyll synthesis.

### 3.2. Revealing the L-Galactose Pathway as the Primary AsA Biosynthetic Pathway in Rapeseed

**Figure 3:**
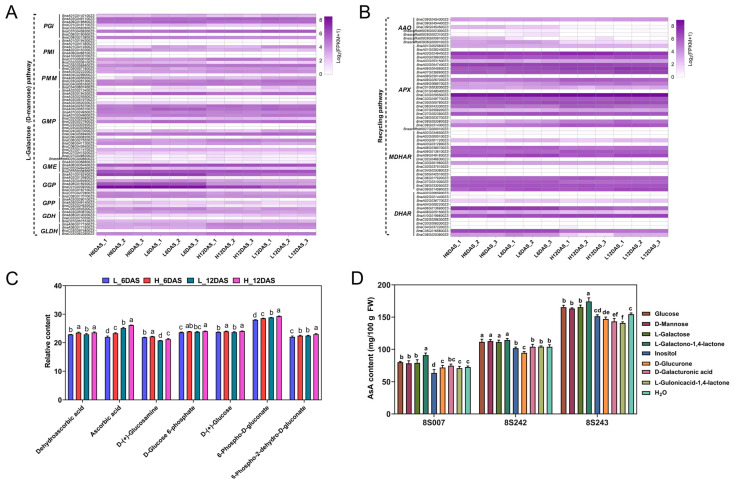
Comprehensive analysis of gene expression, metabolites, and substrate feeding related to AsA in rapeseed seedlings. (**A**) Expression levels of genes related to AsA synthesis in the L-galactose pathway. *PGI*: Glucose-6-phosphate isomerase; *PMI*: Mannose-6-phosphate isomerase; *PMM*: Phosphomannomutase; *GMP*: GDP-D-mannose pyrophosphorylase; *GME*: GDP-mannose-3,5-epimerase; *GGP*: GDP-L-galactose phosphorylase; *GPP*: L-galactose-1-phosphate phosphatase; *GDH*: L-galactose dehydrogenase; *GLDH*: L-galactose-1,4-lactone dehydrogenase; (**B**) Expression levels of genes related to AsA synthesis in the recycling pathway. *AAO*: Ascorbate oxidase; *APX*: Ascorbate peroxidase; *DHAR:* Dehydroascorbate reductase; *MDHAR*: Monodehydroascorbate reductase. (**C**) DAMs in the AsA synthesis pathway identified between high- and low-AsA pools. (**D**) Comparison of AsA content in rapeseed seedlings of three accessions by adding substrates related to four AsA synthesis pathways. The different letters above the bars denote significance groupings (*p* < 0.05) as determined by ANOVA. FW, fresh weight.

A total of 196 genes associated with were found in the ZS11 genome using the AsA-related genes from *A. thaliana* [51] as reference templates (Table S3). Within the four AsA pools, we determined and contrasted the expression levels of these genes implicated in all known AsA biosynthesis and recycling pathways. (Table S4). Among them, we discovered that the L-galactose synthesis (Figure 3A) and the recycling pathways (Figure 3B) had relatively high expression levels of genes connected to AsA. Conversely, the expression levels of genes in the L-gulose, Myo-inositol, and D-galacturonate pathways were found to be relatively lower compared to other pathways (Figure S3). Metabolome analysis identified six DAMs, including dehydroascorbic acid, D-(+)-Glucosamine, D-Glucose-6-phosphate, D-(+)-Glucose, 6-Phospho-D-gluconate, and 6-Phospho-2-dehydro-D-gluconate that were linked to the L-galactose and recycling pathways of AsA biosynthesis (Figure 3C). We further explored the synthesis pathway of AsA in rapeseed by feeding various substrates. After treating rapeseed seedlings with substrates from the L-galactose pathway, which included glucose, D-mannose, L-galactose, and L-galactose-1,4-lactone, the AsA content increased by 5% to 25% in comparison to the control (Figure 3D). However, the AsA content of rapeseed seedlings that were treated with substrates of L-glucose pathway (L-Gulonicacid-1,4-lactone), inositol pathway (inositol, D-Glucurone) and D-galactoacid pathway (D-Galacturonic acid), did not demonstrate a significant increase. Combined with substrate feeding, transcriptome, and metabolome analyses, revealed that the L-galactose pathway is the primary pathway for AsA synthesis in rapeseed.

### 3.3. Effect of Photoperiods on AsA Biosynthesis in Rapeseed

**Figure 4:**
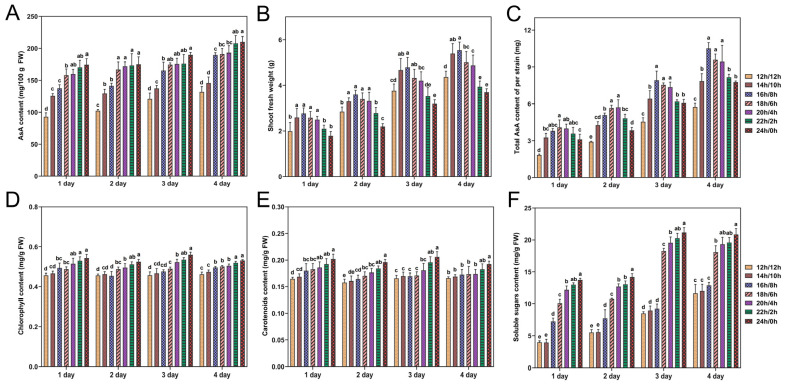
Changes in AsA concentration (**A**), shoot fresh weight (**B**), total AsA content of per strain (**C**), chlorophyll content (**D**), carotenoids content (**E**), and soluble sugars content (**F**) during rapeseed seedlings growth under seven photoperiod treatments. Values and bars represent the means of three replicates ± SD. Different letters indicate significant difference (*p* < 0.05) as obtained by one-way ANOVA test. “1 day, 2 day, 3 day, and 4 day” represents the growth days under different photoperiod conditions. FW, fresh weight.

Metabolome and transcriptome analyses revealed that photosynthesis was one of the main factors regulating AsA biosynthesis in rapeseed. To determine the optimal light photoperiod for high AsA accumulation in rapeseed, different light and dark cycles were employed for rapeseed cultivation in this research. Obviously, the AsA content in rapeseed seedlings increased significantly at 1, 2, 3 and 4 days after the photoperiod treatment, correlating with the extension of the photoperiod (Figure 4A). Compared with the 12 h/12 h (light/dark) (112.0 mg/100 g FW), the average AsA content for the 14 h/10 h (134.6 mg/100 g FW), 16 h/8 h (158.5 mg/100 g FW), 18 h/6 h (172.6 mg/100 g FW), 20 h/4 h (175.1 mg/100 g FW), 22 h/2 h (181.8 mg/100 g FW), and 24 h/0 h (187.4 mg/100 g FW) increased by 20.2% to 67.4% (Figure 4A). However, the shoot fresh weight increased first and then decreased, and reached the highest value at 16 h/8 h, indicating that the continuous extension or reduction in light time was not conducive to the growth of rapeseed seedlings (Figure 4B). By comparing the total accumulation of AsA content (AsA concentration multiplied by biomass) under the seven photoperiods, rapeseed seedlings had the highest AsA accumulation under 18 h/6 h, 20 h/4 h, 16 h/8 h, and 16 h/8 h under day 1, 2, 3 and 4, respectively (Figure 4C), suggesting that the 16 h/8 h photoperiod is the most optimal condition for AsA accumulation in rapeseed seedlings under long-term cultivation. However, before harvest, a proper extension of light time could enhance AsA concentration in rapeseed seedlings without compromising yield. For example, compared with the 16 h/8 h photoperiod, when grown under the 20 h/4 h photoperiod for 24 h and 48 h, the AsA concentration of rapeseed seedlings increased by approximately 16.3% and 21.8%, respectively.

As a significant measure for determining the nutritional worth of vegetables, chlorophyll, carotenoids, and soluble sugars are also highly correlated with photosynthesis [52,53]. As shown in Figure 4D–F, from 12 h/12 h to 24 h/0 h, the patterns of chlorophyll, carotenoids, and soluble sugars were similar to those of AsA concentration, which increased as the light period increased. These findings indicated that continuous light could stimulate the accumulation of chlorophyll, carotenoids, soluble sugars, and AsA in rapeseed seedlings, which has an important effect on the rapeseed seedlings’ development, metabolism, as well as the accumulation of nutrients.

### 3.4. Effect of Photoperiods on the Contents of ROS-Related Indicators and Key Enzyme Activities

**Figure 5:**
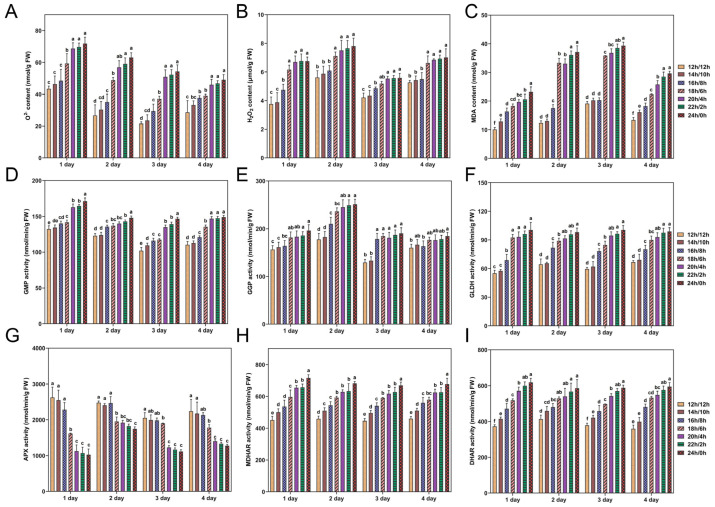
Changes in ROS-related indicators and key enzyme activities during rapeseed seedlings growth under different photoperiod treatments. (**A**) O^2−^ content. (**B**) H_2_O_2_ content. (**C**) MDA content. (**D**) GMP activity. (**E**) GGP activity. (**F**) GLDH activity. (**G**) APX activity. (**H**) DHAR activity. (**I**) MDHAR activity. Values and bars represent the means of three replicates ± SD. Different letters indicate significant difference (*p* < 0.05) as obtained by one-way ANOVA test. FW, fresh weight.

Although light signals are fundamental for plant growth and development, excessive light exposure will inevitably affect photosynthesis and produce excessive ROS, leading to photo-inhibition and photo-oxidative damage of photosynthesis [54,55,56]. To investigate photo-oxidative stress induced by light of different photoperiods, the contents of ROS-related indicators (O^2−^, H_2_O_2_, and MDA) were measured and analyzed. Figure 5A showed that the O^2−^ content in rapeseed seedlings increased with prolonged light exposure during the photoperiod. The photoperiods of 20 h/4 h, 22 h/2 h, and 24 h/0 h showed no significant variation in O^2−^ content. In addition, the trends of MDA, H_2_O_2_, and O^2−^ under different photoperiod treatments were similar (Figure 5B,C). In rapeseed seedlings, different photoperiod treatments resulted in a high positive correlation between O^2−^ and H_2_O_2_ content (r = 0.56, *p* < 0.05). The results indicated that rapeseed seedlings may experience increased oxidative damage under extended illumination. Our findings underscore the significance of maintaining an appropriate photoperiod to promote rapeseed seedlings’ healthy growth and reduce oxidative damage.

Key enzyme activity has a major impact on AsA biosynthesis, which we examined by investigating the L-galactose pathway enzymes GMP, GGP, and GLDH, along with the AsA recycling pathway enzymes APX, MDHAR, and DHAR, under different photoperiod treatments. In rapeseed seedlings, GMP activity increased with prolonged light exposure compared to the 12 h/12 h photoperiod (Figure 5D). At photoperiods of 14 h/10 h, 16 h/8 h, 18 h/6 h, 20 h/4 h, 22 h/2 h, and 24 h/0 h, the average GMP activity increased by 2.7% to 31.5% over four days. Similarly, GGP and GLDH activities in rapeseed seedlings also increased with extended light time compared to the 12 h/12 h photoperiod, with GGP average activity increasing by 3.3% to 32.0% (Figure 5E) and GLDH activity increasing by 3.4% to 61.8% over the same photoperiod (Figure 5F). Among the photoperiods of 20 h/4 h, 22 h/2 h, and 24 h/0 h, GGP and GLDH activities showed no significant variation.

Figure 5G showed a negative correlation between APX activity in rapeseed seedlings and the light time in photoperiod. APX activity was highest in seedlings grown at 12 h/12 h and lowest at 24 h/0 h throughout the experimental period. Compared to the 12 h/12 h, the average APX activity decreased by 2.9% to 45% in other photoperiod treatments. There were no substantial differences in APX activities among the photoperiods of 12 h/12 h, 14 h/10 h, and 16 h/8 h. The temporal changes in the activities of MDHAR and DHAR, as well as their activity differences under various photoperiods, were notably similar (Figure 5H,I). Compared to the 12 h/12 h, MDHAR and DHAR activities in other photoperiods increased by 11.1% to 51.2%, and 11.1% to 56.6%, respectively. These findings were important for understanding how rapeseed seedlings adapted to different photoperiods by regulating enzyme activity.

### 3.5. PCA and Correlation Analysis of AsA and Compositions Under Different Photoperiods

**Figure 6:**
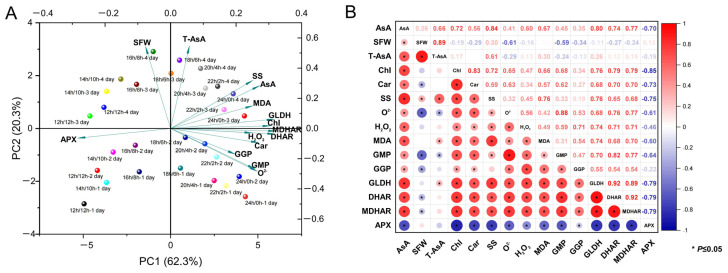
PCA (**A**) and correlation (**B**) analysis of AsA-related physiological parameters under different photoperiods. SFW, shoot fresh weight. T-AsA, total accumulation of AsA content, Chl, chlorophyll, Car, carotenoids, SS, soluble sugars.

PCA of AsA and its compositions in rapeseed seedlings was performed with two principal components. As shown in Figure 6A, PC1 and PC2 represented 62.3% and 20.3% of the overall variability, respectively, successfully segregating the different photoperiods into four distinct groups. Almost all physiological parameters, including AsA, total accumulation of AsA content (T-AsA), chlorophyll (Chl), carotenoids (Car), soluble sugars (SS), H_2_O_2_, O^2−^, MDA, GME, GGP, GLDH, DHAR, and MDHAR, were positively related to PC1, while the shoot fresh weight (SFW) and APX were negatively linked to PC1. The photoperiodic points were distinctly separated along PC1. In addition, the left quadrant featured 12 h/12 h, 14 h/10 h, and 16 h/8 h, whereas the right quadrant comprised 18 h/6 h, 20 h/4 h, 22 h/2 h, and 24 h/0 h. The points of processing days of photoperiod were separated along with PC2. The lower quadrant contained an index on day 1 and day 2 under different photoperiods, while the upper quadrant included an index on day 3 and day 4. The PCA conducted in this study elucidated the physiological indicators in rapeseed seedlings under varying photoperiods, as well as the interaction between enzyme activities and AsA accumulation, which provided a foundation for the informed selection of photoperiods and light treatment durations for AsA biosynthesis.

The correlation coefficients between AsA content and various indicators were presented in Figure 6B. The results revealed that under different photoperiod treatments, a strong positive relationship existed between AsA content and the levels of soluble sugars, chlorophyll, and carotenoids (r = 0.56–0.84, *p* < 0.05). In addition, the content of AsA exhibited significant correlations with the contents of H_2_O_2_, O^2−^, and MDA (r = 0.41–0.67, *p* < 0.05). The activities of GMP, GGP, GLDH, DHAR, and MDHAR showed a strong positive association with AsA (r = 0.35–0.80, *p* < 0.05), with GLDH exhibiting the strongest correlation and GGP the weakest correlation to the level of AsA. Conversely, the activities of APX displayed a significant negative association with AsA (r = −0.70, *p* < 0.05). Furthermore, chlorophyll and carotenoids showed a significant positive correlation with each other (r = 0.83, *p* < 0.05), and the activities of MDAHR and DHAR highlighted a significant positive relationship (r = 0.92, *p* < 0.05). Combined with substrate feeding, enzyme activity identification, and environmental factor regulation, this study provided an effective way for high biosynthesis of AsA in rapeseed seedlings.

### 3.6. Effects of Photoperiod on Gene Expressions in AsA Biosynthesis and Recycling Pathways

**Figure 7:**
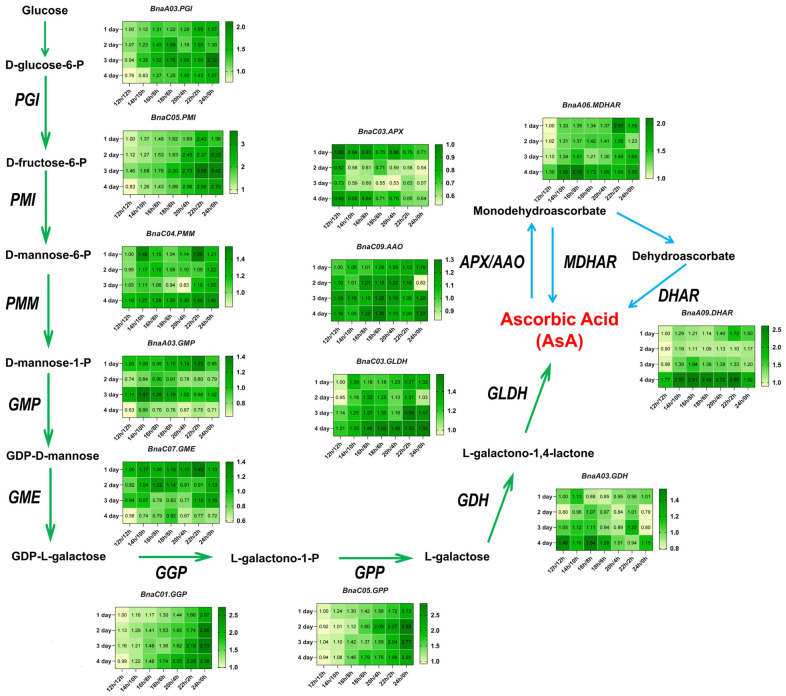
The pathways of L-galactose and recycling of AsA and related gene expression levels during rapeseed seedlings growth under different photoperiods. Values are shown as means ± SD (n = 3). The green arrows indicated the L-galactose pathway in the AsA synthesis route. The blue arrow indicated the recycling pathway in the AsA synthesis route.

To investigate the impact of photoperiod on the molecular regulatory mechanism of dynamic changes in AsA biosynthesis in rapeseed, the transcription levels of 13 key genes with high FPKM values (Table S5) in the L-galactose and recycling pathways were analyzed. As depicted in Figure 7, in the L-galactose pathway of AsA biosynthesis, a comparison between the 12 h/12 h photoperiod shows that the transcript levels of *BnaA03.PGI*, *BnaC05.PMI*, *BnaC01.GGP*, and *BnaC05.GPP* increased with prolonged light exposure. Notably, *BnaC05.PMI* showed the greatest level of expression among the genes analyzed. Within the recycling pathway for AsA synthesis, as the duration of light exposure increased, a decline in the expression level of *BnaC03.APX* was observed, but the expression of *BnaA06.DHAR* and *BnaA09.MDHAR* genes did increase. In summary, the regulatory patterns of key genes implicated in both the L-galactose and recycling pathways in rapeseed seedlings indicate that the regulation of AsA levels is responsive to alterations in photoperiod.

The relationships between AsA concentrations and the transcription levels of key genes under different photoperiod treatments are presented in Figure S4. The analysis revealed a significant positive association between AsA content and the gene expression of several genes, specifically *BnaA03.PGI*, *BnaC05.PMI*, *BnaC01.GGP*, *BnaC05.GPP*, *BnaC03.GLDH*, *BnaA09.MDHAR*, and *BnaA06.DHAR* (r = 0.53–0.75, *p* < 0.05). The expression of *BnaC03.APX* exhibited a negative relationship with AsA (r = −0.46, *p* < 0.05). Additionally, the expressions of *BnaA03.PGI* and *BnaC05.PMI* indicated a positive correlation with each other (r = 0.70, *p* < 0.05). Similarly, *BnaC01.GGP* and *BnaC05.GPP* exhibited a significant positive relationship (r = 0.92, *p* < 0.05), as well as between *BnaA09.MDHAR* and *BnaA06.DHAR* (r = 0.76, *p* < 0.05). Furthermore, the expressions of *BnaA03.PGI*, *BnaC05.PMI*, *BnaC01.GGP*, and *BnaC05.GPP* were negatively correlated with the expression of *BnaC03.APX* (r = −0.50–−0.64, *p* < 0.05). These findings not only enhance our understanding of how photoperiod affects AsA metabolism but also provide a scientific foundation for improving the antioxidant capacity and environmental adaptability of rapeseed seedlings through genetic enhancement. The co-expression patterns among these AsA-related genes and the regulatory network analysis for AsA biosynthesis provided information on target genes for further bioenhancement of AsA content in rapeseed.

## 4. Discussion

### 4.1. Effects of Substrates Related to L-Galactose Pathway and Photoperiod Regulation on AsA Biosynthesis in Rapeseed

Rapeseed, as a novel vegetable, is an excellent source of AsA, with higher content in its seedlings and flower stalks than most vegetables, which will potentially become one of the primary dietary sources of AsA. It is important to comprehend the regulation and biosynthesis of AsA, which is essential for improving its content in fruits and vegetables [57]. In many plants, including Arabidopsis and tomato, the L-galactose pathway is considered the predominant pathway [58]. Through substrate feeding, transcriptome, and metabolome analysis, this study also proved the L-galactose pathway serves as the major route for AsA production in rapeseed seedlings. In rapeseed seedlings, feeding glucose, D-mannose, L-galactose, L-galactose-1,4-lactone, and other substrates related to the L-galactose pathway can greatly increase AsA levels (Figure 3C). Previous studies have also shown that AsA content in *Arabidopsis* plants can be increased by feeding related substrate in the L-galactose pathway [59]. Further, the porphyrin metabolism pathways associated with photosynthesis were enriched in both transcriptome and metabolome. Previous studies have reported that the impact of light on AsA levels has been investigated across a range of plant species, including lettuce, tomato, eggplant, apple, and kiwifruit [11,26,60,61]. Our research results revealed that the AsA levels in rapeseed seedlings increased by 20.2% to 67.4% when the light/darkness time increased from 12 h/12 h to 24 h/0 h (Figure 4A). With the continuous increase in light treatment time, the photoperiod of 16 h/8 h was more conducive to the increase in fresh weight in rapeseed seedlings, resulting in an advantage in total AsA accumulation (Figure 4C). However, when rapeseed seedlings are shifted to a 24 h/0 h photoperiod for 4 days before harvest, the AsA content can exceed 200 mg/100 g fresh weight, significantly surpassing the AsA levels found in other cruciferous vegetables (such as cauliflower, Chinese cabbage, collards, Chinese flowering cabbage, mustard spinach, white cabbage, turnip greens et al.) [62]. Thus, the AsA content in rapeseed seedlings can be enhanced by appropriately extending the lighting duration without adversely affecting yield. This innovative approach not only maximizes the health benefits of rapeseed but also provides a versatile framework for biofortification strategies that can be applied across a range of vegetables and fruits.

### 4.2. Continuous Light Promoted ROS Accumulation and Contributed to the Enrichment of Antioxidants

Prolonged exposure can lead to several negative impacts on plants, including chlorosis of leaves, damage to the photosynthetic apparatus, and accelerated leaf aging [63,64]. In addition to these physiological impacts, prolonged light exposure can disrupt the circadian rhythms of plants, resulting in a misalignment between internal biorhythms and external light conditions [65]. This circadian asynchrony can intensify stress responses and adversely affect plant growth and development. In this research, we found that the levels of ROS-related indicators (H_2_O_2_, O^2−^, and MDA) in the rapeseed increased with the extension of light time (Figure 5A–C). These findings are similar to earlier research, which indicates that elevated light intensity results in a rise in ROS generation [26], resulting in a decrease in biomass. However, many studies have reported the beneficial impacts of continuous light, such as speeding up the reproductive cycle and enhanced quality [28]. The results of our study suggest that extended periods of light exposure enhance carotenoids, chlorophyll, and soluble sugars, reflecting the favorable outcomes of continuous light (Figure 4D–F). For example, carotenoid acts as accessory pigments in photosynthesis and participate in the xanthophyll cycle to prevent photo-oxidative damage [52]. These pigments are essential for photoprotection and also highlight the importance of carbohydrates in regulating AsA levels. Additionally, soluble sugar has an important correlation with the stress resistance of plants, which maintains the integrity of cell membranes by regulating the osmotic potential of plant tissues [66]. The increase in ROS and MDA levels may indicate damage to excessive light energy. However, the concurrent upregulation of photoprotective pigments and soluble sugars suggests an adaptive response to mitigate photo-oxidative stress [67]. These adaptive mechanisms are crucial for maintaining cellular integrity and metabolic homeostasis under prolonged light conditions. This indicates that appropriate stress is conducive to the accumulation of antioxidant nutrients in rapeseed and other crops, which has important significance for improving the commodity value of vegetables with quality as an important evaluation index.

### 4.3. Key Enzyme and Gene Responses to Different Photoperiods in AsA Synthesis

Furthermore, the photoperiod regulates AsA levels by regulating the expression and function of genes and enzymes involved in its biosynthesis and recycling pathways. Our results indicated that the enzymes GMP, GGP, and GLDH, which play crucial roles in the L-galactose pathway, exhibited increased activity with extended light exposure (Figure 5D–F). This response may be a strategic response by the plant to increase AsA production and antioxidant capacity. In chloroplasts, APX can use AsA to reduce H_2_O_2_ to water and form MDHA [68]. Conversely, the activity of APX decreased with increasing light cycle time (Figure 5G), suggesting a shift in the plant’s antioxidant strategy under prolonged light conditions, potentially reallocating resources to other defense mechanisms. In addition, it has been noted that the elevation of AsA during stress may be partially attributed to the heightened decrease rates of MDHA and DHA, processes facilitated by MDHAR and DHAR [68]. This investigation revealed that DHAR and MDHAR activities increased with prolonged photocycle time (Figure 5H,I), consistent with the above conclusions. This indicates that the regeneration of AsA is sensitive to light availability and that increased light may impair the rapeseed’s ability to maintain AsA levels, thus affecting its antioxidant defense. A dynamic regulatory mechanism is suggested by the differential transcription of key genes in the L-galactose and AsA recycling pathways in response to changing light conditions. This is consistent with previous studies demonstrating that light plays a crucial role in regulating AsA biosynthesis by modulating the expression of key genes in the L-galactose pathway [69]. The upregulation of *BnaA03.PGI, BnaC05.PMI, BnaC04.PMM*, *BnaC01.GGP*, *BnaC05.GPP*, and *BnaC03.GLDH* with extended light duration suggests an enhancement in the flux through the L-galactose pathway, potentially contributing to increased AsA production (Figure 7). In this study, our research conclusion is consistent with previous results. Previous research has shown that light influences AsA production in chives by affecting the expression of *GGP* and *GME* [69]. Additionally, it was observed that the expressions of *GMP*, *GPP*, *GLDH*, and *GGP* exhibited fluctuations that corresponded to the variations in the content of the AsA pool in the leaves during both light and dark phases [24]. In the AsA recycling pathway, the decrease in *BnaC03.APX* expression with prolonged light cycles, coupled with the increase in *BnaA09.DHAR* and *BnaA06.MDHAR*, suggests a shift towards more efficient AsA regeneration, which is crucial for sustaining the plant’s antioxidant capacity. The expressions of *BnaA03.PGI, BnaC05.PMI, BnaC04.PMM*, *BnaC01.GGP*, *BnaC05.GPP*, and *BnaC03.GLDH* was negatively correlated with the expression of *BnaC03.APX*. The main reason for this phenomenon may be that APX plays a central role in the plant ascorbic acid-glutathione cycle, protecting plant cells from oxidative damage by scavenging H_2_O_2_ and maintaining AsA regeneration [70]. When APX activity diminishes, the rate of AsA oxidation is reduced, thereby allowing more AsA to be conserved and promoting its accumulation within cells. This suggests a possible regulatory mechanism to maintain metabolic balance. The observed correlations between AsA content and gene expression levels highlight the complex regulatory network governing AsA metabolism in response to changes in photoperiod.

## 5. Conclusions

Our results showed that the L-galactose pathway was identified as the primary route for AsA synthesis in rapeseed leaves, regulated predominantly by light. Rapeseed seedlings exhibited optimal growth and AsA accumulation under a 16 h/8 h photoperiod, with extended light exposure before harvest further enhancing AsA concentration without compromising yield. However, prolonged light exposure also increases oxidative stress, as indicated by elevated ROS production and lipid peroxidation. Notably, appropriate photoperiods can mitigate this oxidative damage, highlighting the need for a balance between light exposure and oxidative stress management. Enzymes critical for AsA biosynthesis, including GMP, GGP, GLDH, DHAR, and MDHAR, are upregulated under increased light conditions, while APX activity shows a negative correlation with AsA content. This underscores the complex interplay between AsA metabolism and photoperiod regulation. Additionally, gene expression patterns in AsA biosynthesis and recycling pathways are modulated by light exposure. Correlation and PCA analyses further elucidate the relationship between AsA content and various physiological parameters under different photoperiods. These findings enhance our understanding of the metabolic mechanisms underlying light-regulated AsA synthesis during rapeseed growth. The results provide valuable insights for optimizing AsA levels in greenhouse-grown rapeseed and lay the foundation for the development of high-value rapeseed products.

## Data Availability

Data will be made available on request.

## Supplementary Materials

The following supporting information can be downloaded at: https://www.mdpi.com/article/10.3390/antiox14020160/s1, Figure S1: Comparison of the relative expression abundance measured by qRT-PCR and RNA-seq. Figure S2: Correlation analysis among the samples based on transcriptomic data. Figure S3: Expression levels of genes related to AsA synthesis in four AsA pools in the L-gulose pathway, Myo-inositol pathway and D-galacturonate pathway. Figure S4: Pearson’s correlation coefficients among the AsA content and genes expressions under different photoperiod treatments. Table S1: Primers of relative genes for quantitative real-time PCR. Table S2: The statistical summary of total clean reads of the 12 samples. Table S3: AsA-related genes reported in *A. thaliana* and homologs identified in *B. napus* ZS11. Table S4: Gene expression information for 196 AsA-related genes. Table S5: Gene expression information for 13 AsA-related genes for L-galactose(D-mannose) and recycling pathways.
