# Supplementary material for: Effect of Photoperiod on Ascorbic Acid Metabolism Regulation and Accumulation in Rapeseed (Brassica napus L.) Seedlings
Source: Antioxidants (Basel). 2025 Jan 29;14(2):160. doi: 10.3390/antiox14020160 (PMC11851679; doi:10.3390/antiox14020160)
Supplement: Supplementary file 1 [file antioxidants-14-00160-s001.zip › Supplementary Figures S1-S4.pdf]

Supporting information

Figure S1. Comparison of the relative expression abundance measured by qRT-PCR and RNA-seq.

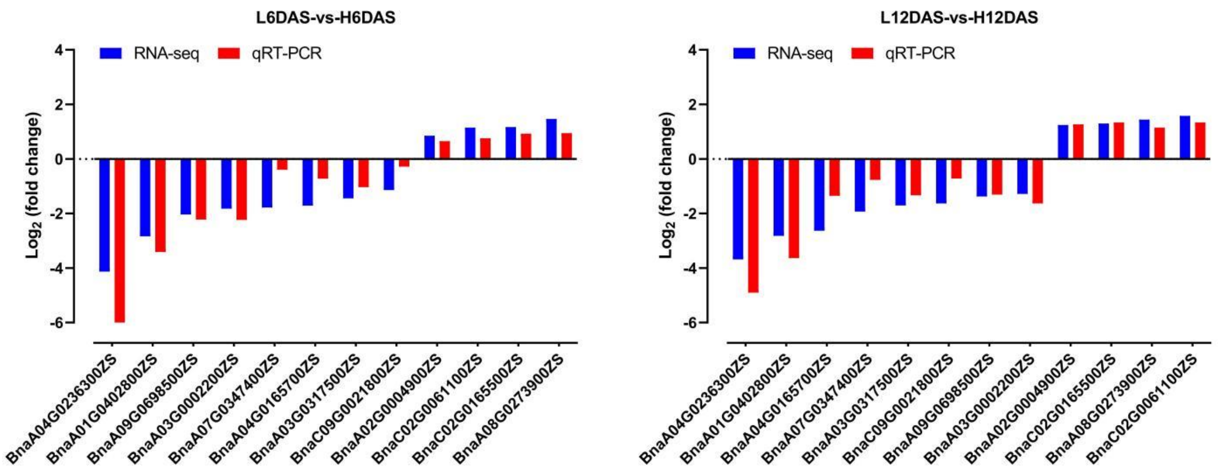

Figure S2. Correlation analysis among the samples based on transcriptomic data.

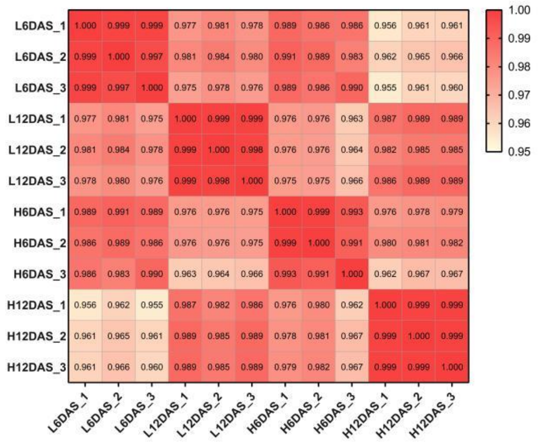

Figure S3. Expression levels of genes related to AsA synthesis in four AsA pools in the L-gulose pathway, Myo-inositol pathway and D-galacturonate pathway.

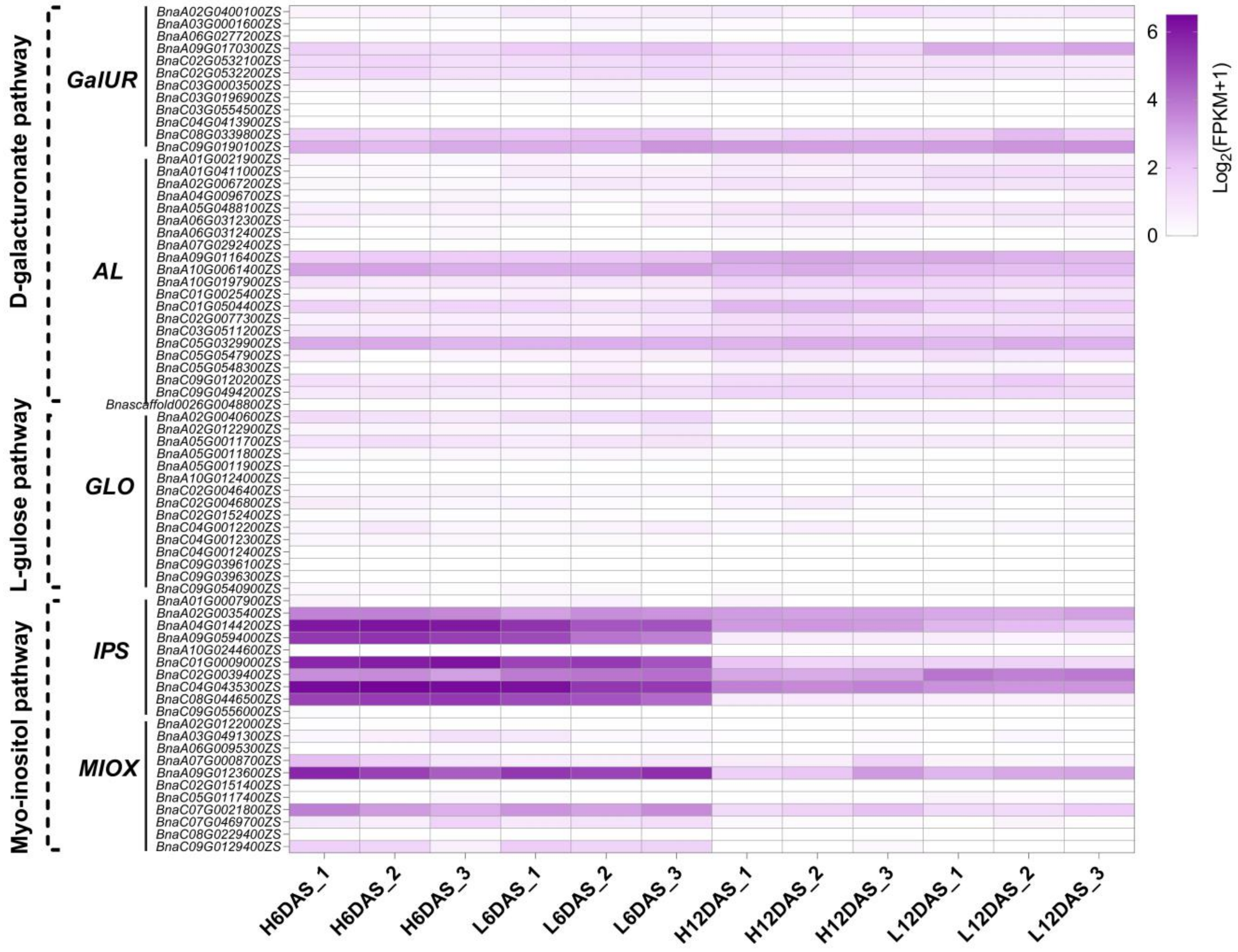

AL: Aldono lactonase; *GalUR*: D-Galacturonate Reductase; *GLO*: Gulonolactone oxidase; *IPS*: L-myo-inositol 1-phosphate synthase; *MIOX*: Myo-inositol oxygenase.

Figure S4. Pearson's correlation coefficients among the AsA content and genes expressions under different photoperiod treatments.

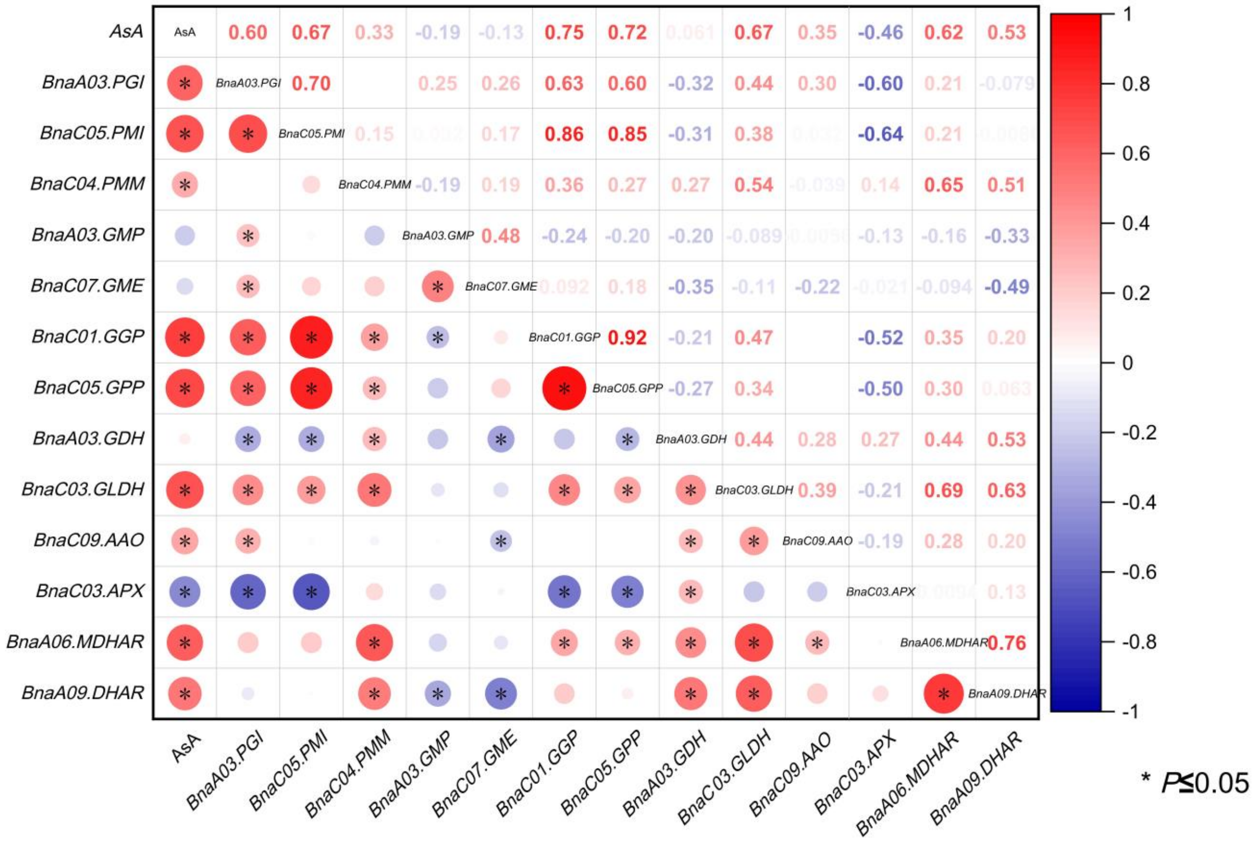

PGI: Glucose-6-phosphate isomerase; *PMI*: Mannose-6-phosphate isomerase; *PMM*: Phosphomannomutase; *GMP*: GDP-D-mannose pyrophosphorylase; *GME*: GDP-mannose-3,5-epimerase; *GGP*: GDP-L-galactose phosphorylase; *GPP*: L-galactose-1-phosphate phosphatase; *GDH*: L-galactose dehydrogenase; *GLDH*: L-galactose-1,4-lactone dehydrogenase; *MDHAR*: Monodehydroascorbate reductase; *DHAR*: Dehydroascorbate reductase; *AAO*: Ascorbate oxidase; *APX*: Ascorbate peroxidase.
